# Racism as a determinant of health: a protocol for conducting a systematic review and meta-analysis

**DOI:** 10.1186/2046-4053-2-85

**Published:** 2013-09-23

**Authors:** Yin Paradies, Naomi Priest, Jehonathan Ben, Mandy Truong, Arpana Gupta, Alex Pieterse, Margaret Kelaher, Gilbert Gee

**Affiliations:** 1 Centre for Citizenship and Globalization, Faculty of Arts and Education, Deakin University, 221 Burwood Highway, Melbourne, Victoria 3125, Australia; 2 McCaughey VicHealth Centre for Community Wellbeing, Melbourne School of Population and Global Health, University of Melbourne, Level 5, 207 Bouverie Street, Melbourne, Victoria 3010, Australia; 3 Oppenheimer Center for Neurobiology of Stress, David Geffen School of Medicine, UCLA, 10833 Le Conte Avenue, Los Angeles CA, USA; 4 Faculty of Education, Monash University, Building 6 Wellington Road, Melbourne, Victoria 3800, Australia; 5 Centre for Health Policy Programs and Economics, Melbourne School of Population and Global Health, University of Melbourne, Level 4, 207 Bouverie Street, Melbourne, Victoria 3010, Australia; 6 Department of Community Health Sciences, UCLA Fielding School of Public Health, P.O. Box 951772, Los Angeles CA, USA

**Keywords:** Racism, Racial discrimination, Mental health, Physical health, Meta-analysis, Systematic review

## Abstract

**Background:**

Racism is increasingly recognized as a key determinant of health. A growing body of epidemiological evidence shows strong associations between self-reported racism and poor health outcomes across diverse minority groups in developed countries. While the relationship between racism and health has received increasing attention over the last two decades, a comprehensive meta-analysis focused on the health effects of racism has yet to be conducted. The aim of this review protocol is to provide a structure from which to conduct a systematic review and meta-analysis of studies that assess the relationship between racism and health.

**Methods:**

This research will consist of a systematic review and meta-analysis. Studies will be considered for review if they are empirical studies reporting quantitative data on the association between racism and health for adults and/or children of all ages from any racial/ethnic/cultural groups. Outcome measures will include general health and well-being, physical health, mental health, healthcare use and health behaviors. Scientific databases (for example, Medline) will be searched using a comprehensive search strategy and reference lists will be manually searched for relevant studies. In addition, use of online search engines (for example, Google Scholar), key websites, and personal contact with experts will also be undertaken. Screening of search results and extraction of data from included studies will be independently conducted by at least two authors, including assessment of inter-rater reliability. Studies included in the review will be appraised for quality using tools tailored to each study design. Summary statistics of study characteristics and findings will be compiled and findings synthesized in a narrative summary as well as a meta-analysis.

**Discussion:**

This review aims to examine associations between reported racism and health outcomes. This comprehensive and systematic review and meta-analysis of empirical research will provide a rigorous and reliable evidence base for future research, policy and practice, including information on the extent of available evidence for a range of racial/ethnic minority groups

## Background

### Introduction

Racism is increasingly recognized as a key determinant of health [1-3]. Racism constitutes phenomena that result in avoidable and unfair inequalities in power, resources and opportunities across racial or ethnic groups. It can be expressed as beliefs, stereotypes, prejudices or discrimination and can range from open threats and insults to phenomena deeply embedded in social systems and structures [4]. Racism can occur at multiple levels, including: internalized (that is, the incorporation of racist attitudes, beliefs or ideologies into one’s worldview), interpersonal (interactions between individuals) and systemic (for example, the racist production, control and access to labor, material and symbolic resources within a society) [4,5].

The contribution of racism as a social determinant of health is receiving growing attention [6]. Racism is thought to affect health through a number of pathways: (1) limited access to social resources such as employment, housing and education and/or increased exposure to risk factors (such as unnecessary contact with the criminal justice system); (2) negative affective/cognitive and other pathopsychological processes; (3) allostatic load and other pathophysiological processes; (4) reduced engagement with healthy behaviors (for example, exercise) and/or increased adoption of unhealthy behaviors (for example, substance misuse) either directly as stress coping or indirectly via reduced self-regulation; (5) direct physical injury caused by race-based violence [2,7-11].

**Figure 1:**
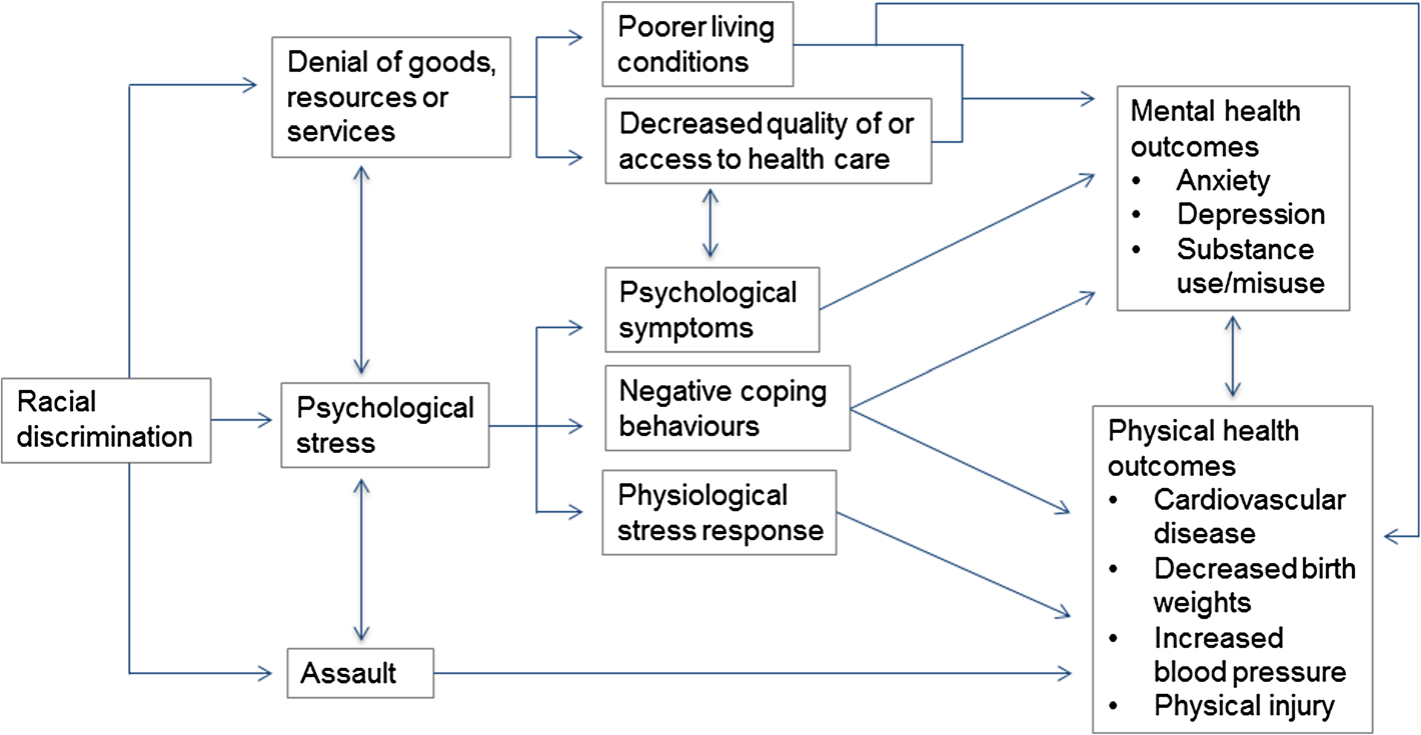
**Pathways between racism and health**[13]**.**

While a number of conceptual diagrams depicting these pathways exist in the current literature [5,8,9,12], in Figure 1 we present a model highlighting the multiple pathways through which racism can affect health. Rather than a comprehensive account of the pathways whereby racism may be related to health, this model will guide our meta-analysis, based on empirical evidence within the current literature.

### Previous reviews

A number of existing literature reviews have focused on racism and health, including four meta-analyses [9,14-16], which have focused on specific population groups, national contexts or broader exposures (that is, discrimination more generally). Previous reviews and meta-analyses have found associations between racism (frequently operationalized as self-reported experiences of racial discrimination) and a range of adverse health outcomes [2,10,11]. Early reviews focused on adverse mental and physical health outcomes in the USA (for example, Williams *et al*. [17]; Brondolo *et al*. [18]; Williams *et al*. [19]; Wyatt *et al*. [20]), finding evidence of consistent associations between racism and mental health outcomes while associations between racism and physical health outcomes were mixed. These reviews were mostly small scale, focused on African-Americans and predominantly on negative physical and mental health. A comprehensive meta-analysis focused on racism and health outcomes has not yet been published.

A systematic review by Paradies [2] identified 138 international empirical population-based studies of self-reported racism and a wide range of health outcomes, finding that the strongest and most consistent associations existed between racism and negative mental health and health-related behaviors. Williams and Mohammed [3] published a comprehensive review of studies conducted between 2005 and 2007 across various settings and population groups. Reviewing 115 studies on perceived racial discrimination and health, they found a consistent relationship between discrimination and a wide range of health outcomes, particularly poor mental health status. Another comprehensive review by Pascoe and Smart Richman, combined with a meta-analysis [9] examined 192 studies on the association between perceived discrimination (not restricted to racial discrimination but with 66% of identified studies focusing on this topic) and mental and physical health. In all, 134 of these studies were included in the meta-analysis, which showed that discrimination had a significant negative effect on both mental and a somewhat weaker but still significant negative association with physical health.

Several reviews and meta-analyses were conducted recently on racism and racial discrimination experienced by specific population groups, such as Asian-Americans, African-Americans as well as children and young people from various racial/ethnic groups. Gee *et al*.’s [11] review of 62 empirical studies on racial discrimination and health outcomes among Asian-Americans found a consistent relationship between racial discrimination and mental health problems but less consistent relationships between racial discrimination and physical and behavioral problems. Lee and Ahn conducted 2 meta-analyses, the first involving 23 studies of Asian-Americans [15] and another involving 51 studies of Latinos in the USA [16]. Both meta-analyses found a strong correlation between racial discrimination and poor mental health. Pieterse *et al*.’s [14] meta-analysis examined associations between perceived racism and mental health in 66 studies of Black Americans, and found a positive association between racism and distress. Priest *et al*.’s [10] systematic review focused on children and young people. Examining 121 studies, they found that negative mental health was the most consistently associated with racial discrimination while other outcomes such as positive mental health, behavior problems and well-being showed mostly significant associations as well.

### Rationale

Previous reviews and meta-analyses of the association between racism and health have had several limitations to their scope. In most reviews and all meta-analyses directly on this topic, participants were from particular population groups and/or national contexts, and the study outcomes examined were restricted to specific health outcome categories, such as mental health (see Table 1 for a summary of previous meta analysis main inclusion criteria). (The only meta-analysis to have used a more general framework, Pascoe and Smart Richman [9], examined discrimination more broadly and did not report racism/racial discrimination separately from other types of discrimination). No previous meta-analyses have examined the magnitude of the associations between racism and health outcomes across all existing studies nor compared the differential effects of racism across a broad range of health outcomes. Neither the magnitude of associations between racism and health across participant subgroups (for example, based on age, gender, race/ethnicity) nor dose-response associations between racism and health outcomes have yet been meta-analyzed for the greater sample of studies specifically on this topic.

**Table 1:** Inclusion criteria used in previous meta-analyses and the current study (participants, exposures, and outcomes)

**Study**	**Participants**	**Exposures**	**Outcomes (authors’ original categorizations)**
Pascoe and Smart Richman [9]	Not restricted	Discrimination (including racial discrimination)	Physical health (risk factors related to cardiovascular disease (for example, blood pressure, intramedial thickness, plaque, and heart rate variability), a multitude of diseases and physical conditions (for example, hypertension, cardiovascular disease, pelvic inflammatory disease, diabetes, yeast infections, and respiratory conditions), other general indicators of illness (for example, nausea, pain, and headaches), and general health questionnaires)
			Mental health (symptomatology scales for mental illness (for example, depressive symptoms, anxiety symptoms, posttraumatic stress symptoms, and indicators of psychosis or paranoia), psychological distress, and indicators of general well-being (for example, well-being, self-esteem, positive self-perceptions, life satisfaction, perceived stress, anger, positive and negative affect, happiness, perceived quality of life, and general mental health))
			Health behaviors (alcohol use and abuse, smoking behavior, substance use, good health habits (for example, sleep, diet, exercise, medication adherence, missing doctor appointments, and eating behaviors and attitudes))
			Stress response (cardiovascular reactivity, anger, psychologically felt stress, changes in state self-esteem, changes in feelings of well-being and life satisfaction, feelings of depression and anxiety, and self-reported positive and negative emotion)
Lee and Ahn [15]	Asian participants (not restricted by country)	Racism and racial discrimination	Mental health (anxiety, depression, psychological distress (including overall measures of mental health))
Lee and Ahn [16]	Latina/o /Hispanic Americans in the USA	Discrimination (including racial discrimination)	Mental health (anxiety, depression, psychological distress (including overall measures of mental health), and unhealthy behaviors (general health behaviors, alcohol and substance use, and perceived physical health)
Pieterse *et al*. [14]	Black American adults in the USA	Racism and racial discrimination	Mental health (anxiety, depression, psychiatric symptoms, life satisfaction, self-esteem, general distress)
Current meta-analysis	Not restricted	Racism and racial discrimination	Physical health (infectious disease and chronic conditions and markers, for example, body mass index (BMI), waist to hip ratio (WHR), blood pressure, metabolic and cardiovascular disease)
			Pregnancy and birth outcomes (for example, premature birth, low birth weight)
			Health behaviors/risk behaviors (for example, alcohol, tobacco, substance use)
			Negative mental health (for example, depression, psychological distress, stress, anxiety, social and emotional difficulties)
			Positive mental health (for example, self-esteem, self-worth, resilience)
			General health (for example, feeling unhappy, feeling unhealthy)
			Well-being, life satisfaction, quality of life
			Healthcare use, satisfaction with healthcare system (for example, use of screening tests, access to healthcare and treatment, adherence to treatment)

Moreover, no previous reviews and/or meta-analyses have examined studies using longitudinal designs separately from and in comparison to non-longitudinal designs. Cross-sectional studies have obvious limitations regarding the temporal ordering of variables and causal inference. This limitation is particularly relevant for our topic: not only may racism cause illness but illness may cause one to perceive and report racism [21]. Accordingly, it is critical to fill the gap in the literature by evaluating the longitudinal associations between racism and health/illness, so as to better understand the causal direction.

Additionally, a focus on longitudinal studies will also explore questions of etiology. Several longitudinal studies have suggested that the association between racism and mental illness appears after a short latency period, whereas the association between racism and physical illness only appears after a longer latency period [21,22]. What this implies is that some of the studies finding null associations between racism and physical health may arise because the studies did not include a long enough etiological period; this further suggests that cross-sectional studies may be biased towards type 2 error in relation to physical outcomes.

The current review and meta-analysis will constitute the most comprehensive research on this topic to date. An extensive systematic search strategy employing broad inclusion criteria (described below) will be used to identify relevant studies and reduce the potential for reporting biases.

### Aims

This systematic review and meta-analysis will examine the key characteristics of studies focusing on reported racism and health, including: (1) where and when studies have been conducted, the racial/cultural/ethnic background, age and gender of study populations, study designs, sample sizes, and data sources used; (2) if racism is defined, how exposure to racism is measured in terms of method of administration, timeframes of exposure as well as targets and perpetrators of racism; (3) the magnitude of associations between reported racism and health in overall and across various outcomes, including a comparison between the differential effect of racism on outcomes from the following categories: physical health, pregnancy and birth outcomes, health behaviors/risk behaviors, negative and positive mental health, general health and well-being, and healthcare use; (4) the magnitude of associations between reported racism and health in longitudinal designs separately from and in comparison to non-longitudinal designs, including change in magnitude of association over time; and (5) the magnitude of associations between reported racism and health and participant subgroups (for example, racial/ethnic groups, national contexts, gender, age groups), and the extent to which associations are consistent across groups, including a subgroup comparison between minority and majority groups.

### Hypotheses

Based on previous reviews and meta-analyses, the general hypotheses that guide our meta-analysis include: (1) the overall effect of racism on health across health outcomes will be significant, and racism will be significantly associated with increased risk of physical illness and mental illness, poor health behaviors and increased risk behaviors, poor pregnancy and birth outcomes, poor general health and well-being, and low healthcare use; (2) the association with racism will generally be stronger among studies employing mental health compared to physical health and other outcomes; (3) with regards to physical health outcomes, racism will show a more robust association (that is, larger effect size, smaller standard errors) among longitudinal studies with longer follow-up periods compared to longitudinal studies with shorter follow-up periods or with cross-sectional studies; and (4) the effect of racism on health outcomes will be consistently statistically significant across racial/ethnic minority and majority groups, national contexts, gender groups and age groups.

While previous reviews and meta-analyses provide some evidence supporting these hypotheses among specific groups and in particular contexts, this proposed review and meta-analysis will allow a more comprehensive and quantitative assessment of the evidence for these associations across the whole body of existing studies focused on racism and health.

## Methods and design

### Design

The research will consist of a systematic review and a meta-analysis. The systematic review will follow the reporting guidelines and criteria set in Preferred Reporting Items for Systematic Reviews (PRISMA) [23].

### Criteria for considering studies

#### Types of studies

We will include published and unpublished empirical studies that examine the relationship between racism and health outcomes. Only studies using quantitative methods and reporting quantitative data will be included. These may include: cross-sectional, cohort (prospective and retrospective), case control, experimental, and intervention designs (randomized controlled trials, cluster randomized controlled trials, and non-randomized controlled studies, before and after studies, interrupted time series studies). We will exclude from the meta-analysis any studies that do not report empirical associations between racism and health. Further, studies with inappropriate and/or insufficient data to allow meta-analysis will be documented, but excluded from the analysis.

#### Types of populations

All age groups and participants from any racial, ethnic, cultural or religious group will be included.

#### Exposure measures

While acknowledging that measurement of racism is a complex and developing field, this review will focus on reported racism as the exposure. This will include racism self-reported by individuals, proxy reports (for example, a child’s experiences of racism as reported by the child’s carer) and experiences of vicarious racism (for example, witnessing racism experienced by family or friends). Measures of exposure to racism use different retrospective timeframes (for example, 1 month prior to measurement, 12 months prior to measurement). All exposure timeframes will be included. Studies that report only results from broader measures of discrimination, wherein the specific effect of racism cannot be isolated, will be documented but excluded from the meta-analysis.

#### Outcome measures

Guided by key outcomes identified in previous systematic reviews in the field, the following health outcomes will be included: (1) physical health (infectious disease and chronic conditions and markers for example, body mass index (BMI), waist to hip ratio (WHR), blood pressure, metabolic and cardiovascular disease); (2) pregnancy and birth outcomes (for example, premature birth, low birth weight); (3) health behaviors/risk behaviors (for example, alcohol, tobacco, substance use); (4) negative mental health (for example, depression, psychological distress, stress, anxiety, social and emotional difficulties); (5) positive mental health (for example, self-esteem, self-worth, resilience); (6) general health (for example, feeling unhappy, feeling unhealthy); (7) well-being, life satisfaction, quality of life; and (8) healthcare use, satisfaction with healthcare system (for example, use of screening tests, access to healthcare and treatment, adherence to treatment).

### Identification of eligible studies and data extraction

#### Search strategy

The search will be conducted in English and include studies from the earliest time available to the present. Studies in languages other than English will be excluded from the review. For a list of terms that will be used, please see Additional file 1. The following databases and electronic collections will be searched: Medline, PsycInfo, Sociological Abstracts, Social Work Abstracts, ERIC, CINAHL, Academic Search Premier, Web of Science, ProQuest (for dissertation/theses). Reference lists will be manually searched for relevant studies. In addition, Google, key websites, book chapters and personal contact with experts will also be included in the search [24].

#### Selection of studies

Search results will be imported into Endnote X5 [25], duplicates deleted, and two reviewers will independently screen all titles and abstracts in order to assess for eligibility for inclusion. Full texts of potentially eligible studies will be obtained when required to assist screening for final inclusion. Any discrepancies between reviewers during the screening process with regard to the inclusion/exclusion of studies will be resolved by consensus and/or by discussion with a third reviewer. Rationale for study exclusion will be recorded as part of the screening process [24].

#### Data extraction

Data from included studies will be extracted into an Excel spreadsheet independently by two reviewers, with data then compared and inconsistencies resolved by consensus and/or by a third reviewer. Some studies appear in multiple publications. Data from these studies will all be recorded, but we will use the data from the same study only once during our analysis stage (that is, studies will not be ‘double counted’). Data to be extracted will include [26]: authors; year of publication; type of publication; study years; study design (including sampling procedure); definition of racism; exposure measure(s), including tool/instrument names and author(s), number of items, psychometric properties (focusing on internal consistency measures), method of administration, type of report (self, proxy, vicarious), exposure timeframe, targets and perpetrators of racism; outcome measure(s), including outcome category and subcategory, tools/instruments name, units of measurement, number of items, psychometric properties (focusing on internal consistency measures), outcome timeframe (when reported retrospectively); timepoints (for measurement of outcomes when reported more than once); subgroups (when outcomes are reported separately for subgroups); sample characteristics, including sample size (including subgroup sample size when applicable), study location (country/nation) and participant demographics such as age, racial/ethnic/cultural background, gender, religion, education, income, socioeconomic status and migration status.

Study findings will include: unadjusted strength and direction of associations between self-reported racism and health outcomes, including associations as reported separately for different participant subgroups (that is, subgroup analyses), control and treatment groups, severity of the exposure to racism (that is, analyses of dose-response), and different timepoints; extracted measures will include sample size, test results and the type of data used, for example, correlation coefficient, standardized beta regression coefficients, odds ratios and standardized mean differences.

The strength and direction of associations in studies that adjust for covariates that may influence these associations will also be reported. For each study, the results for the most extensive adjusted model will be reported. Extracted measures will include sample size, test results and the type of data used.

## Study quality and critical appraisal

### Quality assessment

Studies included in the review will be appraised for quality using Critical Appraisal Skills Programme (CASP) tools tailored to each study design [27].

### Analysis

Data that meet all inclusion criteria will first be summarized descriptively and then analyzed statistically. Data will be analyzed using the statistical software Comprehensive Meta Analysis (CMA) Version 2 [28]. Based on the available measures of association, ways of collapsing and the metric to be used will be determined. For example, if correlation coefficients are used, other statistical measures (for example, regression coefficients with standard deviations, odds ratios, dichotomous measures such as the χ^2^ test) will be converted to correlation coefficients, with unadjusted odds ratios converted to correlation coefficients using the formula suggested in Digby [29] (see also Pascoe and Smart Richman [9]) and standardized mean differences converted using the CMA software [28]. Correlation coefficients, odds ratios, and Cohen’s D will be most relevant to employ as measures of effect. *P* values and 95% confidence intervals (CIs) will also be reported. Analyses of the association between racism and health will be conducted for different health outcome measures, and should sufficient longitudinal data be reported, for different timepoints and different racial/ethnic/cultural subgroups. Heterogeneity of effect sizes will then be assessed among studies that focus on similar outcomes using the Q and I^2^ statistics. Using the CMA, weighted effect sizes will be calculated to account for variation in sample sizes, thus giving more weight to effects from larger samples. We anticipate that a random-effects model will be used in aggregating effect sizes. This model is more appropriate than a fixed-effects model given our aim to generalize our findings to the population of studies on racism and health outcomes (Hedges and Vevea [30]; see also Pieterse *et al*. [14] for using a similar approach). Mixed effect models will be used for the moderator analyses, allowing for the testing of differences between levels of study characteristics (for example, study design, study quality). Sensitivity analyses and additional subgroup analyses will be conducted, for example with regard to age and gender.

### Bias assessment

Three methods will be used to assess publication bias among the sample of studies. First, we will produce funnel plots and examine their symmetry. Second, we will use Egger’s weighted regression method. Third, we will calculate a failsafe *N*, to estimate the number of unlocated studies with an average zero effect size required to change the results substantively (for a similar use of these methods, see Pieterse *et al*. [14]). Another possible measure of bias involves a comparison of mean effect sizes between published and unpublished sources (see [9]). Should a publication bias be detected, we intend to use Duval and Tweedie’s [31,32] trim and fill method to estimate and adjust for missing (not reported) studies.

## Discussion

We anticipate that racism will negatively influence health with magnitude of effect varying by study characteristics, subgroups as well as health outcomes.

### Strengths and limitations of the review

Strengths of this review include clear definitions and inclusion criteria, and a transparent systematic approach to searching, screening and reviewing studies as well as extracting data using standardized forms and duplication at all stages. Our search area is large enough and our inclusion criteria broad enough to encompass a wide range of exposure measures of racism and racial discrimination as well as health outcomes, and is likely to identify and synthesize current evidence in the field in order to inform future research as well as policy and practice. As much as possible, by using data reported in existing studies, this review will provide comprehensive statistical analyses not previously available. Although every effort will be made to locate unpublished studies, our findings may still be vulnerable to selective reporting. Another limitation of the review is the inclusion of studies published in English only. Despite a predefined systematic approach to screening and reviewing that includes assessment of inter-rater reliability, the study will also involve judgments made by review authors, which can result in bias.

### Dissemination

Findings will be disseminated in peer-reviewed publications, conference presentations as well as within publicly available fact sheets and evidence summaries produced in conjunction with review authors’ academic institutions and policy and practice partners.

## Competing interests

The authors declare they have no competing interests.

## Authors’ contributions

YP conceived and designed the study. NP assisted with overall design and preparation of the study protocol and led development of the search strategy. JB primarily developed screening and data extraction methods. MT contributed to developing the background and search strategy. MK contributed to the background and search strategy. AP and AG provided advice on statistical analysis. MK and GG contributed to the background, search strategy and statistical analysis. All authors read and approved the final manuscript.

## Supplementary Material

Additional file 1 Search strategy. Click here for file
