# Supplementary material for: Racism as a determinant of health: a protocol for conducting a systematic review and meta-analysis
Source: Syst Rev. 2013 Sep 23;2:85. doi: 10.1186/2046-4053-2-85 (PMC3850958; doi:10.1186/2046-4053-2-85)
Supplement: Additional file 1 — Search strategy. [file 2046-4053-2-85-S1.pdf]

Additional file 1: Search Strategy

MEDLINE Search Strategy (adapted for other databases as needed)

Terms:

1 birth* (ts)

2 gestation* (ts)

3 health* (ts)

4 well-being (ts)

5 wellbeing (ts)

6 disease* (ts)

7 illness* (ts)

8 BMI (ts)

9 “body mass index” (ts)

10 WHR (ts)

11 “waist hip ratio (ts)

12 anthropometric* (ts)

13 “blood pressure” (ts)

14 hypertension (ts)

15 cardiovascular (ts)

16 overweight (ts)

17 obes* (ts)

18 depressi* (ts)

19 anxi* (ts)

20 distress (ts)

21 stress (ts)

22 suicid* (ts)

23 sleep* (ts)

24 (social* OR behav* OR emotio* OR develop* OR psych*) (AB) AND (difficul* OR problem* OR delay* OR adjust*) (ts)

25 self-esteem (ts)

26 “self esteem” (ts)

27 “life satisfaction” (ts B)

28 “quality of life” (ts B)

29 resilien* (ts)

30 alcohol (ts)

31 tobacco (ts)

32 smok* (ts)

33 “substance use” (ts)

34 drug* (ts)

35 health (ts) AND (care OR service* OR clinic*) (ts)

36 Diseases (MESH)

37 Psychiatry (MESH)

38 Psychology (MESH)

39 1 - 38 (OR)

40 ts=(discrim* OR bias OR prejud* OR hostil* OR harass* OR bully* OR “unfair treat*” OR oppress*) OR mh=prejudice

41 ts=(rac* OR ethnic* OR cultur* OR religio* OR migra* OR immigra* OR refugee*) OR mh=ethnic groups OR mh= minority groups

42 ts=(longit* OR cohort* OR trial* OR “follow up” OR prospective OR retrospective OR “cross section*” OR cross-section* OR intervention* or quantitative or survey* OR “case-control” or “case control” or “randomised control* trial*” or “randomized control* trial*” or ”before and after” or “interrupted time series” or questionnaire* or registr* OR evaluat* or audit*) OR mh=Longitudinal Studies OR mh=Epidemiologic Research Design OR mh=Epidemiologic Study Characteristics as Topic or mh=registries

43 **39 AND 40 AND 41 AND 42**
